# Biatrial and Biventricular Reference Ranges Based on Cardiac Magnetic Resonance in Sickle Cell Disease Patients Without Heart Damage

**DOI:** 10.3390/diagnostics14242816

**Published:** 2024-12-14

**Authors:** Laura Pistoia, Antonella Meloni, Vincenzo Positano, Alessandra Quota, Elisabetta Corigliano, Giuseppe Messina, Stefania Renne, Michela Zerbini, Simona Romani, Gianfranco Sinagra, Lamia Ait Ali, Sophie Mavrogeni, Amalia Lupi, Filippo Cademartiri, Alessia Pepe

**Affiliations:** 1 U.O.C. Ricerca Clinica, Fondazione G. Monasterio CNR-Regione Toscana, 56124 Pisa, Italy; laura.pistoia@ftgm.it; 2 Department of Radiology, Fondazione G. Monasterio CNR-Regione Toscana, 56124 Pisa, Italy; antonella.meloni@ftgm.it (A.M.); positano@ftgm.it (V.P.); fcademartiri@ftgm.it (F.C.); 3 Bioengineering Unit, Fondazione G. Monasterio CNR-Regione Toscana, 56124 Pisa, Italy; 4 Servizio di Talassemia, Ospedale “V. Emanuele III”, 93012 Gela, Italy; alequota@hotmail.com; 5 Ematologia Microcitemia, Ospedale San Giovanni di Dio—ASP Crotone, 88900 Crotone, Italy; annalisacorigliano@alice.it; 6 Centro Microcitemie, Grande Ospedale Metropolitano “Bianchi-Melacrino-Morelli”, 89100 Reggio Calabria, Italy; gspmessina@virgilio.it; 7 Struttura Complessa di Cardioradiologia-UTIC, Presidio Ospedaliero “Giovanni Paolo II”, 88046 Lamezia Terme, Italy; stefania.renne@virgilio.it; 8 Diagnostica per Immagini e Radiologia Interventistica, Ospedale del Delta, 44023 Lagosanto, Italy; m.zerbini@ausl.fe.it; 9 Dipartimento Cardiotoracovascolare, Azienda Sanitaria Universitaria Giuliano Isontina (ASUGI), Università di Trieste, 34128 Trieste, Italy; simonaromani90@gmail.com (S.R.); gianfranco.sinagra@asuits.sanita.fvg.it (G.S.); 10 Istituto di Fisiologia Clinica—CNR, 54100 Massa, Italy; aitlamia@ifc.cnr.it; 11 Onassis Cardiac Surgery Center, 17674 Athens, Greece; sophie.mavrogeni@gmail.com; 12 Istituto di Radiologia, Dipartimento di Medicina, Università di Padova, 35128 Padova, Italy; amalia.lupi@unipd.it

**Keywords:** sickle cell disease, cardiac magnetic resonance, biventricular volumes

## Abstract

Background/Objectives: We aimed to establish biatrial and biventricular reference ranges using cardiac magnetic resonance (CMR) parameters in SCD patients without heart damage. Methods: This study compared CMR parameters, quantified by cine SSFP sequences, in 48 adult SCD patients without apparent cardiac involvement (defined by the absence of known risk factors, normal electrocardiogram, and no macroscopic myocardial fibrosis or significant cardiac iron on T2* CMR) to matched cohorts of 96 healthy controls and 96 thalassemia major (TM) patients without cardiac damage. Nine paediatric SCD patients were also analysed and compared to age- and gender-matched groups of nine TM patients and nine healthy subjects. Results: In all groups, studied males displayed higher biventricular volumes and mass indexes than females. Male SCD patients showed significantly higher left ventricular (LV) end-diastolic volume index (EDVI), LV end-systolic volume index (ESVI), LV stroke volume index (SVI), cardiac index, LV and right ventricular (RV) mass index, and atrial areas than healthy subjects. Females with SCD exhibited increased LV EDVI, LV SVI, RV mass index, and left atrial area index compared to healthy controls. SCD and TM patients showed comparable biatrial areas and biventricular volumes and function. When compared to TM, SCD males exhibited a larger mass index, while SCD females showed an increased RV mass index. CMR parameters were similar across all paediatric groups. Conclusions: By establishing the biatrial and biventricular reference ranges through CMR for adult male and female SCD patients, we aimed to prevent possible misdiagnosis of cardiomyopathy in this population by taking into account cardiac adaptation due to anaemia.

## 1. Introduction

Sickle cell disease (SCD) is one of the most commonly inherited haemoglobin disorders, with a prevalence of 1/2000 births in Europe and 1/30 births in central Africa [1,2,3]. Patients with SCD present an autosomal mutation in the gene that encodes the β globin chain (βS), which leads to an abnormal haemoglobin variant that causes red blood cells to become rigid and assume a sickle or crescent shape [4,5,6,7]. The spectrum of SCD includes individuals in the homozygous state (βSS or sickle cell anaemia) as well as various compound heterozygotes, in which the sickle mutation is inherited along with other structural haemoglobin variants, like haemoglobin C or D, or in conjunction with mutations in another globin gene affecting alpha globin, beta globin, or gamma globin [8,9,10,11]. The hallmarks of the disease are chronic severe haemolytic anaemia and the vaso-occlusive crisis caused by sickle-shaped red blood cells, which promote multi-organ damage and are responsible for the cardiovascular complications of SCD [12,13,14,15]. Biventricular dysfunction, pulmonary hypertension, myocardial infarction, and sudden death are mainly observed [16,17,18]. The anaemia is responsible for a compensatory increase in cardiac output and stroke volume. The high-output state leads to biventricular enlargement and hypertrophy [19]. Sometimes, the heart involvement in SCD is characterised by restrictive physiology with diastolic dysfunction, left atrial dilation, and normal systolic function [20,21]. Iron deposition in the myocardium caused by blood transfusions and regional fibrosis are other possible contributing factors to cardiac impairment, even though they are a quite rare finding in SCD patients [22,23,24].

Progress in the management of SCD has resulted in prolonged life expectancy for affected individuals [25]. Consequently, the prevalence of heart disease has risen among older individuals with SCD, with cardiac involvement accounting for nearly one-fourth of deaths in this population [16,26].

An accurate and reproducible method for non-invasively assessing myocardial involvement in patients with hemoglobinopathies is cardiovascular magnetic resonance (CMR) [27,28]. CMR is the gold-standard method to assess biventricular volumes and function with a high reproducibility [29]. Moreover, multiparametric CMR is a key tool for the quantification of myocardial iron burden through the T2 star (T2*) technique and for the evaluation of macroscopic myocardial fibrosis to exclude irreversible myocardial damage [30]. CMR is crucial for the early detection and monitoring of cardiac disease in patients with hemoglobinopathies [31]. The benefits of quantitatively assessing CMR parameters include grading the severity of the disease, monitoring changes during therapy, and evaluating prognosis, which are crucial for efficient management of patients with hemoglobinopathies.

As known from studies on the general population, referring to normal values for quantitative CMR is crucial for distinguishing normal states from disease and for interpreting results [32]. Cardiac function indices in patients with hemoglobinopathies are different from those in the normal population, mainly due to chronic anaemia and, eventually, pre-existing iron burdens [33,34]. For a more accurate diagnostic evaluation, reference ranges for biventricular function parameters were identified in patients with beta thalassemia major (β-TM) [34,35,36]. Establishing appropriate reference ranges for β-TM patients enabled early detection of impaired cardiac function, as iron overload is not strongly correlated with heart dysfunction and a relatively high percentage of β-TM patients show an impairment of cardiac function without significant myocardial iron burden [28]. As several studies have shown significantly greater biventricular dimensions and mass in SCD patients when compared to β-TM [33,34,37,38], reference CMR ranges for patients with SCD could be useful to obtain a tailored and accurate evaluation of the heart in these patients. To our knowledge, no studies have examined if SCD patients without evidence of cardiac damage have different reference ranges for biventricular volumes and function compared to healthy controls.

The aim of this study was to determine the reference ranges for biatrial areas and biventricular volumes and function in SCD patients without myocardial iron overload and cardiac impairment by using CMR.

## 2. Materials and Methods

### 2.1. Study Population

The Myocardial Iron Overload in Thalassemia (MIOT) project was a collaborative Italian network comprising over 60 thalassemia centres and validated magnetic resonance imaging (MRI) sites [30]. The project enrolled patients with hemoglobinopathies consecutively, provided they met the following criteria: (1) adult or paediatric male and female patients with thalassemia syndromes or structural haemoglobin variants requiring MRI to assess cardiac and liver iron burden; (2) written informed consent; (3) written authorisation for use and disclosure of protected health information; and (4) no absolute contraindications to MRI. A centralised web-based database captured patients’ clinical, anamnestic, and instrumental data across participating MIOT centres [30].

Among the 157 SCD patients enrolled in the Myocardial Iron Overload in Thalassemia (MIOT) project, we selected 48 adult patients (36.95 ± 10.91 years, 28 females) who met the following criteria: (1) no cardiovascular risk factors; (2) no history of cardiac disease; (3) normal electrocardiogram; (4) no macroscopic myocardial fibrosis; (5) no myocardial iron overload (all cardiac segments with T2* ≥ 20 ms); and (6) no wall motion abnormalities. Among the selected SCD patients, 31 had a diagnosis of sickle β-thalassemia; a total of 23 patients had received regular blood transfusions or regular exchange transfusions (≥4/year) for at least one year; and 25 patients were non-transfusion-dependent (no or sporadic blood or exchange transfusions).

### 2.2. Control Populations

As a control population, we studied a group of 96 healthy subjects matched 2:1 by age and gender with SCD patients. Control healthy subjects fulfilled the following criteria: (1) no history of cardiac or non-cardiac disease; (2) no cardiovascular risk factors; (3) normal physical examination; (4) normal electrocardiogram; (5) normal echocardiographic examination; and (6) no absolute contraindications to the MRI [39,40].

Moreover, we studied 96 TM patients randomly selected from the MIOT project to perform a 2:1 match by age and gender with SCD patients. TM patients were selected following the same criteria as SCD patients. All TM patients were regularly transfused to maintain a pre-transfusion haemoglobin concentration above 9–10 g/dL. MRI scanning was performed within one week before the scheduled blood transfusion.

### 2.3. Paediatric Population

We also studied a group of 9 paediatric SCD patients enrolled in the MIOT project, and we compared them with 9 healthy subjects and 9 TM patients selected on the basis of a 1:1 match by age and gender. SCD and TM paediatric patients and healthy subjects were selected on the basis of the same criteria as the corresponding adult groups.

The study complied with the Declaration of Helsinki and was approved by the institutional ethics committee. Informed consent was obtained from all patients and healthy subjects for being included in the study.

### 2.4. Magnetic Resonance Imaging (MRI)

MRI exams were performed on 1.5 T scanners (GE Healthcare, Milwaukee, WI, USA; Siemens Healthineers, Erlangen, Germany; Philips, Best, the Netherlands). Signal acquisition was performed using an eight-element cardiac phased-array receiver surface coil with end-expiratory breath holding and ECG-gating.

To quantify biventricular function, steady-state free precession cine sequences were obtained during 8 s breath holds in the vertical and horizontal long-axis planes and in sequential contiguous 8 mm short-axis slices from the atrio-ventricular ring to the apex [35]. Thirty cardiac phases were acquired per heartbeat. To fully encompass the ventricles, 10 to 14 slices were obtained. The most apical slice considered was the first slice that showed no blood pool at the end-diastole. The most basal slice considered was the one that showed a remaining part of the thick myocardium and was below the aortic valve. The sequence parameters were as follows: flip angle 60°, field of view 38 × 38 cm, matrix 256 × 256 pixels.

Image analysis was performed using MASS^®^ software Version 4.2 (Medis, Leiden, The Netherlands). Previous studies have demonstrated high inter- and intra-observer reproducibility of biventricular function measurements in both healthy subjects [41] and patients with hemoglobinopathies [28]. Endocardial and epicardial borders of the wall were manually traced in each slice at both end-diastole and end-systole. Moreover, the papillary muscles and trabeculation within the left ventricular (LV) and right ventricular (RV) cavities were delineated and considered myocardial mass rather than part of the blood pool. End-diastolic and end-systolic volumes (EDV and ESV, respectively) were determined without relying on geometric assumption of the ventricular shape. The mass was calculated as the volume of the myocardium multiplied by its specific weight, which was assumed to be 1.05 g/cm^3^. The estimation of the RV mass was centralised in the coordinating centre of the MIOT Network. A resident (Simona Romani) conducted the analysis under the supervision of an expert operator with over 20 years of experience (A.P.). Biventricular volumes and mass were indexed to the body surface area (BSA), calculated using the variation of the Dubois and Dubois formula [42]. The stroke volume index (SVI) was determined as the difference between the indexed EDV (EDVI) and the indexed ESV (ESVI). The ejection fraction (EF) was calculated as the ratio between the SVI and the EDVI. Left and right atrial areas were estimated from the 4-chamber view projection in the ventricular end-systolic phase.

Myocardial iron overload was assessed using a T2* multiecho multislice approach. This method’s reliability has been previously established [43] and validated through comparison with autopsy findings [44]. Three parallel short-axis views (basal, medium, and apical) of the LV were acquired at nine echo times (TEs) [43]. T2* image analysis was conducted using HIPPOMIOT^®^ (Version 1.0, Consiglio Nazionale delle Ricerche and Fondazione Toscana Gabriele Monasterio, Pisa, Italy) a custom-built previously validated software program [43]. According to the standard American Heart Association (AHA)/American College of Cardiology (ACC) model [45], the software calculated the T2* value for all 16 segments of the LV. A global heart T2* value was determined by averaging these segmental values.

To detect the presence of macroscopic myocardial fibrosis, late gadolinium enhancement (LGE) short-axis images were acquired 10–18 min after intravenous administration of Gadobutrol (Gadovist^®^; Bayer; Berlin, Germany). Also, vertical, horizontal, and oblique long-axis views were acquired. Patients with a glomerular filtration rate <30 mL/min/1.73 m^2^ or those who declined the contrast agent did not undergo the LGE imaging. The presence of LGE was confirmed through visualisation in two different views [46].

### 2.5. Statistical Analysis

All data were analysed using SPSS version 13.0. Continuous variables were summarised as the mean ± standard deviation (SD), and categorical variables were presented as frequencies and percentages. The Kolmogorov–Smirnov test was used to assess the normality of distribution of the continuous variables.

For normally distributed continuous variables, independent samples *t*-test (for 2 groups) or one-way ANOVA (for more than 2 groups) were used for group comparisons. Non-parametric Mann–Whitney U or Kruskal–Wallis tests were applied to non-normally distributed continuous data. Chi-square tests were conducted for categorical variables. To control for multiple comparisons, the Bonferroni correction was applied.

Statistical significance was set at a 2-tailed probability value of 0.05.

## 3. Results

### 3.1. Demographics in Adult SCD Patients, TM Patients, and Healthy Controls

The mean serum ferritin of SCD patients was 1244.97 ± 1258.41 ng/mL, and the mean haemoglobin concentration was 9.47 ± 1.19 ng/mL (9.71 ± 1.48 for the males and 9.29 ± 0.89 for the females). Furthermore, 59.5% of patients were chelated and 63.2% were receiving hydroxyurea therapy. The mean haemoglobin concentration of TM patients was 9.48 ± 0.50 ng/mL, and the mean serum ferritin was 848.24 ± 674.40 ng/mL. No significant difference in mean haemoglobin and ferritin levels was found between SCD and TM adult patients (*p* = 0.154 and *p* = 0.494, respectively).

Table 1 shows the demographics of adult SCD patients, TM patients, and healthy subjects, categorised by gender. The three groups were well-matched for age. The height, weight, and BSA results were significantly different in the three groups in both males and females. Differences in body mass index (BMI) were found only in males. In particular, height and BSA were significantly lower in SCD females than in healthy control females (*p* < 0.0001 and *p* = 0.021, respectively). No significant differences were found in weight and BMI of female SCD patients vs. healthy females. In SCD males, height, weight, and BSA were significantly lower than in healthy males (*p* < 0.0001, *p* < 0.015, and *p* < 0.0001, respectively), while no significant differences were found in BMI.

SCD and TM patients of both genders showed no significant differences in any of the physical characteristics analysed.

Regarding the comparison between TM patients and healthy subjects, we found significantly lower height, weight, and BSA in both males (*p* < 0.0001 for all variables) and females (*p* < 0.0001, *p* = 0.003 and *p* < 0.0001, respectively). Male TM patients also had a significantly lower BMI (*p* = 0.034) than healthy subjects, while no significant differences were found in females.

### 3.2. Comparison of Biatrial and Biventricular CMR Parameters in the Three Groups

**Figure 1:**
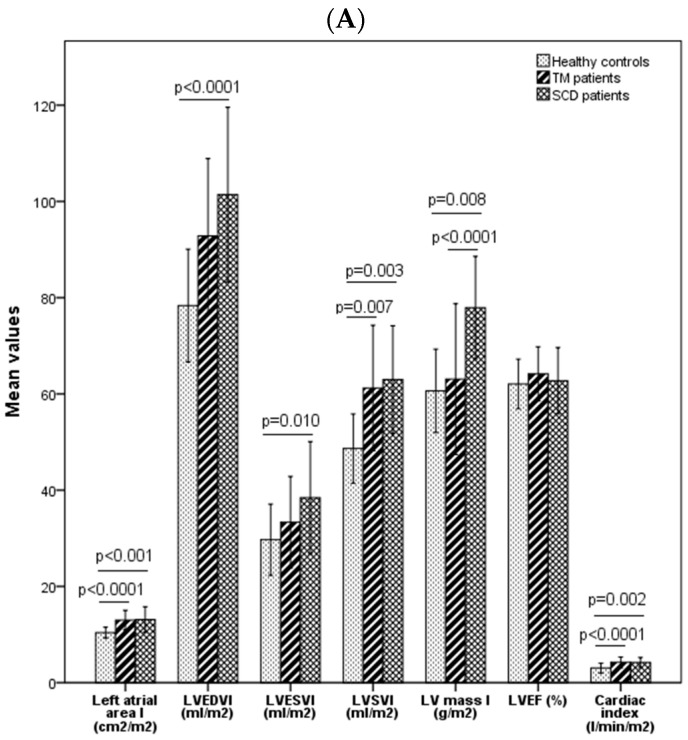

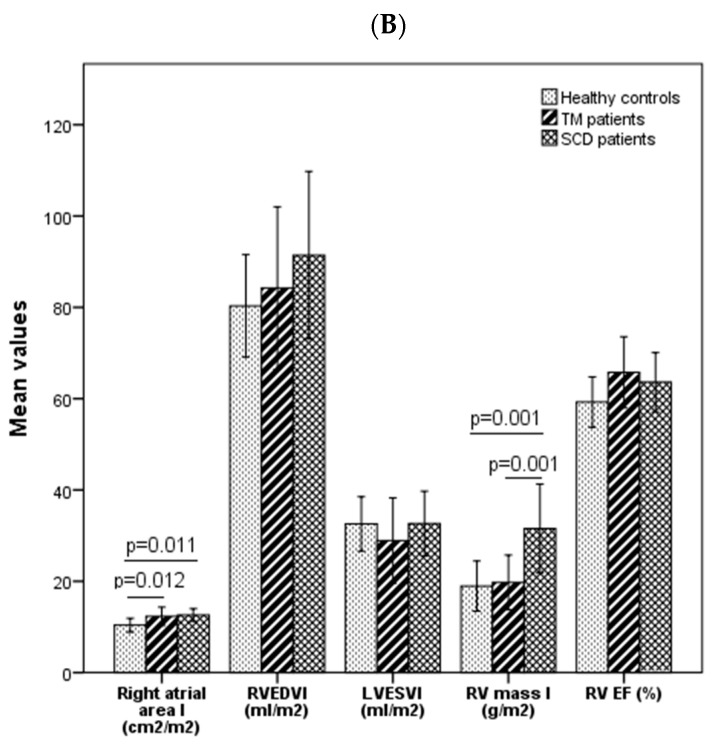
Comparison of biatrial size and biventricular function parameters through CMR among adult SCD, TM, and healthy males (left ventricle parameters (**A**), right ventricle parameters (**B**)). SCD = sickle cell disease, TM = thalassemia major, LV = left ventricular, EDVI = end-diastolic volume index, ESVI = end-systolic volume index, SVI = stroke volume index, EF = ejection fraction, RV = right ventricular. Statistical methods: one way ANOVA, Kruskal–Wallis test (group comparisons); Mann–Whitney U, Bonferroni correction (1-to-1 comparisons).

**Figure 2:**
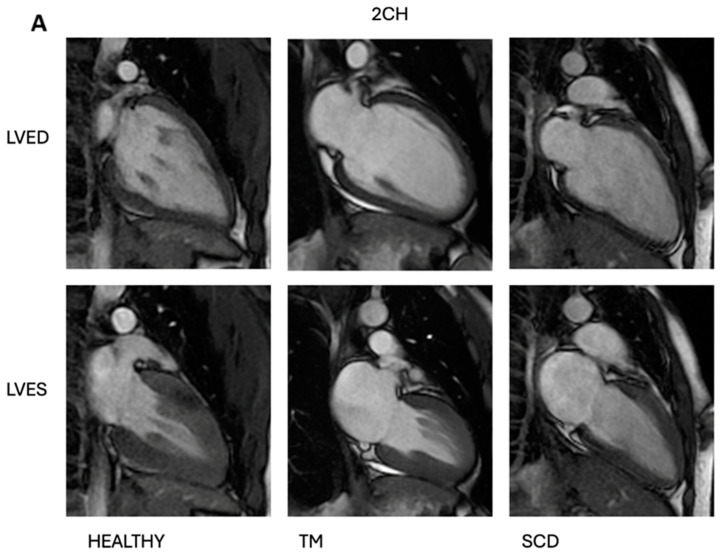

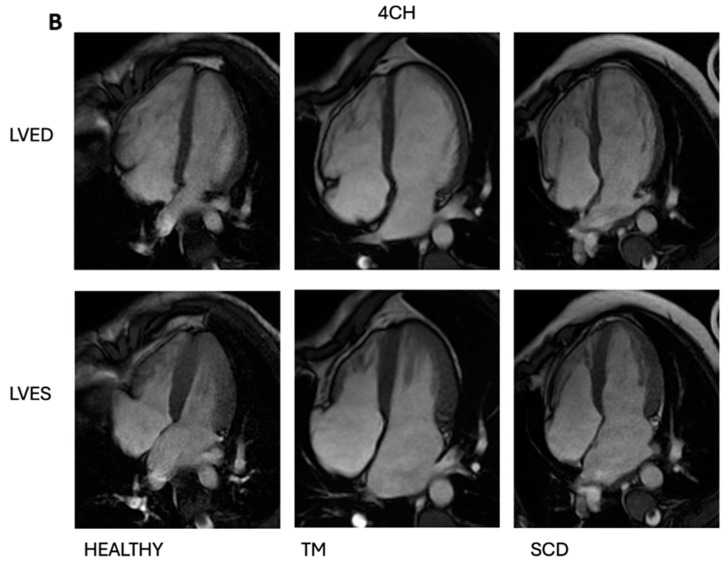
Three males, 18 years old (two-chamber view (**A**) and four-chamber view (**B**). Healthy subject: LVED volume index = 69 mL/m^2^, LVES volume index = 26 mL/m^2^; TM patient: LVED volume index = 88 mL/m^2^, LVES volume index = 38 mL/m^2^; SCD patient: LVED volume index = 139 mL/m^2^, LVES volume index = 64 mL/m^2^. LVED = left ventricular end diastole; LVES = left ventricular end systole; TM = thalassemia major; SCD = sickle cell disease; CH = chamber.

The results of the comparison of biatrial and biventricular parameters for SCD and TM adult males and healthy males are summarised in Figure 1. Left and right atrial areas were significantly different in the three male groups (*p* < 0.0001 and *p* = 0.004, respectively). Both SCD and TM male patients showed a left and right atrial area significantly higher than healthy subjects. No differences in atrial areas were found between SCD and TM patients. LV volume indexes and LV mass index were significantly different among the three groups (LVEDVI: *p* < 0.0001; LVESVI: *p* = 0.012; LVSVI: *p* = 0.001; LV mass index: *p* < 0.0001). Specifically, LV volume indexes and LV mass index were significantly higher in male SCD patients than in healthy subjects (LVEDVI: *p* < 0.0001; LVESVI: *p* = 0.010; LVSVI: *p* = 0.003; LV mass index: *p* = 0.008). Moreover, male TM patients showed a significantly higher LVSVI than healthy controls (*p* = 0.007) and a significantly lower LV mass index than male SCD patients (*p* < 0.0001). The cardiac index was significantly different among the groups (*p* < 0.0001), and no differences were found for LVEF (*p* = 0.286). RV volume indexes were not significantly different among SCD patients, TM patients, and healthy subjects (RVEDVI: *p* = 0.137; RVESVI: *p* = 0.195), while RVEF and RV mass index were significantly different (*p* = 0.046 and *p* = 0.001, respectively). Figure 2 shows an imaging example of the heart of a SCD patient, a TM patient, and a healthy subject (males, 18 years old).

**Figure 3:**
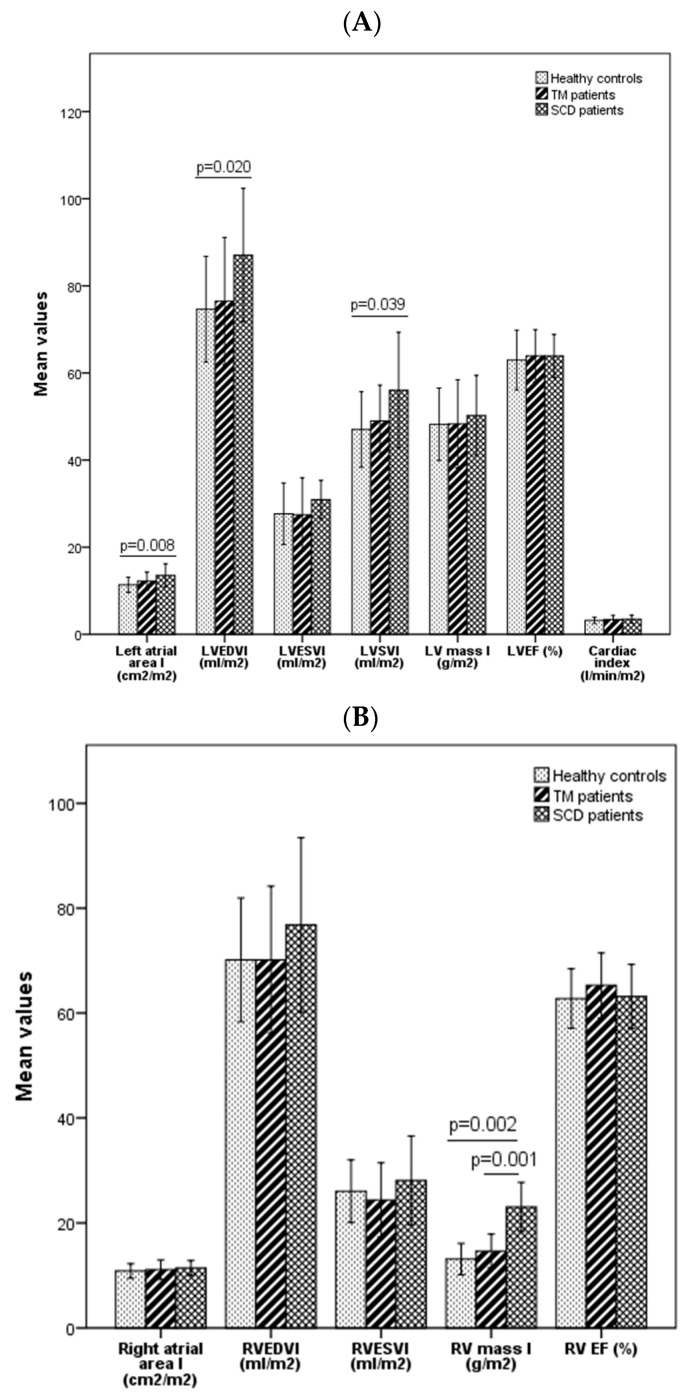
Comparison of biatrial size and biventricular function parameters through CMR among adult SCD, TM, and healthy females (left ventricle parameters (**A**), right ventricle parameters (**B**)). SCD = sickle cell disease, TM = thalassemia major, LV = left ventricular, EDVI = end-diastolic volume index, ESVI = end-systolic volume index, SVI = stroke volume index, EF = ejection fraction, RV = right ventricular. Statistical methods: one way ANOVA, Kruskal–Wallis test (group comparisons); Mann–Whitney U, Bonferroni correction (1-to-1 comparisons).

The biatrial and biventricular parameters for SCD and TM adult females and healthy females are presented in Figure 3. In females, the left atrial area was significantly different among the three groups (*p* = 0.010); in particular, it was significantly different between SCD patients and healthy controls (*p* = 0.008). No significant differences were found among the groups for the right atrial area (*p* = 0.801). LVEDVI and LVSVI were significantly different among female groups (*p* = 0.024 and *p* = 0.038, respectively). Specifically, the LVEDVI and the LVSVI were significantly higher in SCD patients than in healthy controls (*p* = 0.020 and *p* = 0.039, respectively). No significant differences were found among the groups for LVESVI, LV mass index, LVEF, and cardiac index (*p* = 0.205, *p* = 0.375, *p* = 0.878 and *p* = 0.568, respectively). RV volume and function indexes were not significantly different among female SCD patients, TM patients, and healthy subjects (RVEDVI: *p* = 0.423; RVESVI: *p* = 0.753; RVEF: *p* = 0.983). RV mass was significantly different among the three groups (*p* = 0.002). Both SCD and TM female patients showed an RV mass index significantly higher than healthy subjects.

### 3.3. Influence of Gender on Biatrial and Biventricular Parameters

Table 2 shows the comparison of MR parameters between sexes in the three groups.

In SCD patients, all biventricular volume indexes and mass indexes as well as the right atrial area index were significantly larger in males than in females. The LV, the RV EF, and the left atrial area index were not different between sexes.

In TM patients, biventricular EDVI, SVI, and mass index were significantly larger in males than in females, while no difference was found in biventricular EF. The right atrial area index was significantly larger in males than in females, while no differences were found in the left atrial area index.

In healthy subjects, all biventricular volume indexes, with the exception of LV SVI, and mass indexes were significantly larger in males than in females. No difference was detected in biventricular EFs or the cardiac index. Females had a left atrial area index significantly larger than males.

### 3.4. Reference Ranges in SCD Patients

Table 3 details the mean biatrial and biventricular MR parameters for adult SCD males and females and the reference ranges defined as mean—2 SD for the volumes and masses and as mean—1 SD for the EF, considering the high cardiac output state in anaemic patients. In case of a non-normal distribution, the log-transformed data were taken into account.

### 3.5. CMR Parameters in Pediatric Patients

Table 4 shows the comparison of demographics and MR function parameters in paediatric SCD patients, TM patients, and healthy subjects. The three groups were well-matched for gender and age (SCD: 9.81 ± 2.73, TM 9.84 ± 2.81, healthy 9.80 ± 2.95 years, *p* = 0.995). No differences in demographic parameters among the three groups were observed. SCD and TM paediatric patients and healthy paediatric subjects did not differ in terms of biatrial and biventricular MR function parameters.

## 4. Discussion

The chronic haemolytic anaemia observed in patients with hemoglobinopathies is responsible for hyperdynamic circulation, characterised by an increase in biatrial and biventricular volumes, lower peripheral resistance, and a high cardiac output [47]. Moreover, as confirmed in this study, patients tend to have a smaller body size than healthy subjects due to poor growth and delayed pubertal development [48,49] and may have myocardial iron overload, although rarely seen in patients with SCD [23]. Therefore, in these patients, reference ranges for biatrial areas and biventricular function parameters are different from ranges in healthy subjects [34], and the use of appropriate reference ranges is important for the early and accurate detection of impaired function and to avoid misdiagnosis of cardiac impairment.

Previous studies that highlighted the importance of using reference ranges were conducted on β-TM patients [34,36]. More recently, appropriate reference ranges for biventricular volumes, EF, and LV mass were established in a large cohort of β-TM patients [35]; the study was conducted on a well-treated cohort of 251 β-TM patients without cardiac damage by using a multiparametric CMR method. Moreover, the reference ranges were normalised to BSA, sex, and age. The study showed that TM patients have different reference ranges for biventricular volumes and function and LV mass compared to healthy controls. To our knowledge, there are no studies that have evaluated the opportunity to identify reference ranges for the SCD patient population.

In the present study, we selected a group of SCD patients with no cardiovascular risk factors or history of cardiac disease, a normal electrocardiogram, no macroscopic myocardial fibrosis, and no myocardial iron overload based on T2* CMR, and we compared them with a group of healthy volunteers and a group of β-TM patients without cardiac damage well-matched by sex and age.

Firstly, we observed that males had higher biventricular volumes and mass indexes than females in all three groups. Gender-based differences in cardiac volumes and mass are well-established and routinely considered in clinical practice. Our finding is in agreement with previous studies on normal subjects [50,51] and TM [35] and SCD patients [38]. For this reason, we conducted a separate analysis for males and females.

Male SCD patients had significantly higher LV volumes, cardiac index, biventricular mass indexes, and biatrial area indexes than healthy subjects. No significant differences were found in biventricular EF. Similar findings were found in previous studies [19,33,52]. In the SCD and healthy female groups, we found less marked differences. This may be due to the potential capacity of females to better tolerate the effects of anaemia [28].

Despite similar biventricular volumes and ejection fractions, when compared to TM, SCD patients showed larger biventricular mass indexes in the male group and a larger RV mass index in the female group. This datum could be explained by the greater lifetime exposure of SCD patients to more severe anaemia and cardiovascular remodelling due to the later starting of transfusions or the absence of a regular transfusion regimen. Previous studies found significant differences between SCD and TM adult patients not only in mass index but also in biventricular dimensions and cardiac function [19,33]. The smaller disparity observed in this study may be due to the similar haemoglobin levels of the two patient groups and the absence of iron overload and myocardial fibrosis, which were used as inclusion criteria for both patient groups. Indeed, in addition to the effects of chronic anaemia alone, other factors could influence cardiac biventricular volumes.

No significant differences in MR parameters were found among the three paediatric groups. This datum is not consistent with the study by Meloni A. et al. [38], in which significant differences between SCD and TM paediatric patients were found. The reason for the different findings could be the younger age of patients in this study. Moreover, the receipt of appropriate treatment since birth by both SCD and TM patients attenuated the differences with healthy subjects.

### Limitations

A limitation of this study is that the presence of endocrine complications or HCV infection, which could influence ventricular function, was not considered as an exclusion criterion. The second limitation is the relatively small sample size of the study population, the adult patients with SCD, which may limit the generalisability of the findings. Furthermore, the paediatric patients included in the study were also a small number, but it is necessary to take into account that SCD is a rare disease. Considering the absence of data in the literature about bi-atrial and biventricular reference ranges based on CMR in SCD patients, our data, even if based on a relatively small population, could contribute to improving the diagnostic accuracy for cardiomyopathy in patients with SCD.

Furthermore, we would like to emphasise one limitation of this manuscript by explaining that the data we used are the only data we could gather and that they originate from one of the largest databases globally concerning this rare disease. Although the data are derived from a single database, which, while large, may limit the applicability of the findings to other populations, we have conducted such a study and are convinced of its value.

The cross-sectional design of this study limits our understanding of the longitudinal trajectory of these parameters. Further prospective studies are needed to investigate how these parameters evolve with disease progression or treatment.

## 5. Conclusions

In conclusion, the present study showed that SCD adult patients without cardiac damage have significantly higher biatrial and biventricular MR parameters than age- and gender-matched healthy subjects and a significantly higher mass index than TM patients. To interpret abnormal cardiac conditions, it is essential to know the reference ranges of cardiac structure and function due to the hemoglobinopathy. Based on the different reference ranges observed in SCD patients without cardiac damage, the identification of specific thresholds may improve their clinical care. The use of appropriate cut-offs for biatrial and biventricular MR parameters for adult males and females with SCD could be important to enhance diagnostic accuracy for the detection of cardiomyopathy, while accounting for the adaptation due to anaemia, thus avoiding misdiagnosis.

## Figures and Tables

**Table 1:** Comparison of demographics among SCD patients, TM patients, and healthy controls.

	SCD Patients	TM Patients	H Controls	*p* Value	1-to-1 Comparisons *p* Value
Females	*N* = 28	*N* = 56	*N* = 56		
Height (m)	159.6 ± 5.9	157.5 ± 8.0	165.8 ± 6.9	<0.0001	SCD vs. H: *p* < 0.0001 SCD vs. TM: *p* = 0.68 TM vs. H: *p* < 0.0001
Weight (kg)	58.3 ± 6.8	57.9 ± 11.3	63.5 ± 12.5	0.004	SCD vs. H: *p* = 0.43 SCD vs. TM: *p* = 1.00 TM vs. H: *p* = 0.003
BMI (kg/m^2^)	22.9 ± 2.4	22.9 ± 3.3	23.1 ± 4.1	0.96	
BSA (m^2^)	1.6 ± 0.1	1.6 ± 0.2	1.7 ± 0.2	<0.0001	SCD vs. H: *p* = 0.021 SCD vs. TM: *p* = 1.00 TM vs. H: *p* < 0.0001
Males	*N* = 20	*N* = 40	*N* = 40		
Height (m)	170.3 ± 6.3	167.0 ± 5.8	178.1 ± 7.4	<0.0001	SCD vs. H: *p* < 0.0001 SCD vs. TM: *p* = 0.13 TM vs. H: *p* < 0.0001
Weight (kg)	67.5 ± 12.7	63.9 ± 9.0	78.2 ± 11.5	<0.0001	SCD vs. H: *p* < 0.015 SCD vs. TM: *p* = 1.09 TM vs. H: *p* < 0.0001
BMI (kg/m^2^)	23.2 ± 3.5	22.9 ± 2.8	24.7 ± 3.1	0.030	SCD vs. H: *p* = 0.23 SCD vs. TM: *p* = 1.00 TM vs. H: *p* = 0.034
BSA (m^2^)	1.8 ± 0.2	1.7 ± 0.1	2.0 ± 0.2	<0.0001	SCD vs. H: *p* < 0.0001 SCD vs. TM: *p* = 0.40 TM vs. H: *p* < 0.0001

SCD = sickle cell disease, TM = thalassemia major, H = healthy, *N* = number, BMI = body mass index, BSA = body surface area. Statistical methods: one way ANOVA, Kruskal–Wallis test (group comparisons); Mann–Whitney U, Bonferroni correction (1-to-1 comparisons).

**Table 2:** Comparison of biatrial and biventricular MR parameters between sexes.

Adults	Males	Females	*p* Value
SCD patients			
Left atrial area index (cm^2^/m^2^)	13.1 ± 2.7 (*N* = 13)	13.5 ± 2.7 (*N* = 13)	0.69
Right atrial area index (cm^2^/m^2^)	12.6 ± 1.4 (*N* = 13)	11.2 ± 1.6 (*N* = 13)	0.024
LV EDVI (mL/m^2^)	101.5 ± 20.7	85.0 ± 13.4	0.003
LV ESVI (mL/m^2^)	38.9 ± 12.2	30.8 ± 5.9	0.026
LV SVI (mL/m^2^)	62.4 ± 12.3	53.9 ± 11.0	0.016
LV mass I (g/m^2^)	77.7 ± 11.3	50.0 ± 8.3	<0.0001
LV EF (%)	62.1 ± 6.9	63.3 ± 5.6	0.51
Cardiac index (L/min/m^2^)	4.1 ± 1.0	3.5 ± 0.9	0.033
RV EDVI (mL/m^2^)	92.6 ± 17.0	76.2 ± 14.1	0.001
RV ESVI (mL/m^2^)	33.7 ± 6.8	28.0 ± 10.2	0.034
RV mass I (g/m^2^)	29.0 ± 1.4	20.9 ± 7.6	0.010
RV EF (%)	63.0 ± 5.6	64.2 ± 8.1	0.57
TM patients			
Left atrial area index (cm^2^/m^2^)	13.0 ± 2.0 (*N* = 31)	12.2 ± 2.1 (*N* = 48)	0.12
Right atrial area index (cm^2^/m^2^)	12.2 ± 2.1 (*N* = 31)	11.1 ± 2.0 (*N* = 48)	0.019
LV EDVI (mL/m^2^)	90.8 ± 16.9	78.1 ± 15.6	<0.0001
LV ESVI (mL/m^2^)	32.6 ± 9.7	29.1 ± 11.2	0.11
LV SVI (mL/m^2^)	59.8 ± 12.9	49.5 ± 8.3	<0.0001
LV mass I (g/m^2^)	62.8 ± 15.0	48.6 ± 10.2	<0.0001
LV EF (%)	64.4 ± 6.0	63.6 ± 6.1	0.52
Cardiac index (L/min/m^2^)	4.1 ± 1.1	3.5 ± 1.0	0.007
RV EDVI (mL/m^2^)	84.7 ± 17.6	73.5 ± 15.0	0.001
RV ESVI (mL/m^2^)	30.1 ± 10.0	27.0 ± 10.3	0.14
RV mass I (g/m^2^)	19.3 ± 6.2	14.4 ± 3.1	0.001
RV EF (%)	64.3 ± 7.9	64.2 ± 6.3	0.97
Healthy subjects			
Left atrial area index (cm^2^/m^2^)	10.4 ± 1.1 (*N* = 17)	11.4 ± 1.8 (*N* = 28)	0.044
Right atrial area index (cm^2^/m^2^)	10.6 ± 1.6 (*N* = 17)	10.9 ± 1.4 (*N* = 28)	0.49
LV EDVI (mL/m^2^)	82.5 ± 15.1	76.0 ± 12.8	0.025
LV ESVI (mL/m^2^)	30.8 ± 8.7	27.5 ± 7.5	0.045
LV SVI (mL/m^2^)	51.7 ± 9.8	48.6 ± 8.9	0.12
LV mass I (g/m^2^)	66.7 ± 11.8	51.1 ± 8.9	<0.0001
LV EF (%)	62.6 ± 5.9	64.0 ± 7.2	0.33
Cardiac index (L/min/m^2^)	3.2 ± 0.8	3.3 ± 0.8	0.37
RV EDVI (mL/m^2^)	84.0 ± 12.8	72.1 ± 11.5	<0.0001
RV ESVI (mL/m^2^)	33.1 ± 7.9	25.8 ± 6.1	<0.0001
RV mass I (g/m^2^)	19.2 ± 5.3	15.1 ± 5.0	<0.0001
RV EF (%)	60.4 ± 6.9	64.0 ± 6.3	0.008

SCD = sickle cell disease, TM = thalassemia major, *N* = number, LV = left ventricular, EDVI = end-diastolic volume index, ESVI = end-systolic volume index, SVI = stroke volume index, EF = ejection fraction, RV = right ventricular. Statistical methods: *t*-test and Mann–Whitney U test.

**Table 3:** Cut-offs for biatrial and biventricular CMR parameters for adult males and females SCD patients.

	Mean ± SD	Reference Ranges
Males		
Left atrial area index (cm^2^/m^2^)	13.1 ± 2.7	[9.2–18.1]
Right atrial area index (cm^2^/m^2^)	12.6 ± 1.4	[9.8–15.4]
LV EDVI (mL/m^2^)	101.5 ± 20.7	[67.1–147.7]
LV ESVI (mL/m^2^)	38.9 ± 12.2	[19.7–69.8]
LV SVI (mL/m^2^)	62.4 ± 12.3	[37.8–87]
LV mass I (g/m^2^)	77.7 ± 11.3	[57.6–102.8]
LV EF (%)	62.1 ± 6.9	[55.2–69]
Cardiac output (L/min)	7.4 ± 1.9	[5.5–9.3]
Cardiac index (L/min/m^2^)	4.1 ± 1.0	[3.1–5.1]
RV EDVI (mL/m^2^)	92.6 ± 17.0	[58.6–126.6]
RV ESVI (mL/m^2^)	33.7 ± 6.8	[20.1–47.3]
RV mass I (g/m^2^)	29.0 ± 1.4	[26.2–31.8]
RV EF (%)	63.0 ± 5.6	[57.4–68.6]
Females		
Left atrial area index (cm^2^/m^2^)	13.5 ± 2.7	[9.3–19.2]
Right atrial area index (cm^2^/m^2^)	11.2 ± 1.6	[8.0–14.4]
LV EDVI (mL/m^2^)	85.0 ± 13.4	[62.1–113.7]
LV ESVI (mL/m^2^)	30.6 ± 5.9	[20.4–44.6]
LV SVI (mL/m^2^)	53.9 ± 11.0	[31.9–75.9]
LV mass I (g/m^2^)	50.0 ± 8.3	[35.6–68.3]
LV EF (%)	63.3 ± 5.6	[57.7–68.9]
RV EDVI (mL/m^2^)	76.2 ± 14.1	[48–104.4]
RV ESVI (mL/m^2^)	28.0 ± 10.2	[7.6–48.4]
RV mass I (g/m^2^)	20.9 ± 7.6	[5.7–36.1]
RV EF (%)	64.2 ± 8.1	[56.1–72.3]

LV = left ventricular, EDVI = end-diastolic volume index, ESVI = end-systolic volume index, SVI = stroke volume index, EF = ejection fraction, RV = right ventricular.

**Table 4:** Comparison of demographics and CMR parameters in paediatric SCD patients, TM patients, and healthy subjects.

	SCD Patients *N* = 9	TM Patients *N* = 9	Healthy Controls *N* = 9	*p* Value
Height (m)	134.6 ± 12.8	133.1 ± 16.5	140.3 ± 18.3	0.527
Weight (kg)	32.0 ± 11.6	33.7 ± 8.8	34.3 ± 11.3	0.720
BMI (kg/m^2^)	17.1 ± 2.6	18.8 ± 2.1	16.9 ± 1.7	0.081
BSA (m^2^)	1.1 ± 0.2	1.1 ± 0.2	1.1 ± 0.3	0.917
Left atrial area (cm^2^/m^2^)	12.5 ± 2.3	14.4 ± 4.2	12.3 ± 2.8	0.736
Right atrial area (cm^2^/m^2^)	11.9 ± 1.9	12.3 ± 3.5	13.2 ± 0.9	0.141
LV EDVI (mL/m^2^)	92.8 ± 14.1	89.9 ± 15.3	80.6 ± 5.1	0.120
LV ESVI (mL/m^2^)	33.2 ± 7.1	31.7 ± 5.7	28.8 ± 4.5	0.250
LV SVI (mL/m^2^)	59.3 ± 9.0	58.3 ± 10.9	51.8 ± 4.7	0.173
LV mass I (g/m^2^)	54.8 ± 5.1	54.3 ± 14.1	57.3 ± 9.9	0.781
LV EF (%)	63.9 ± 4.5	64.0 ± 3.4	64.1 ± 4.5	0.979
Cardiac output (L/min)	4.9 ± 0.9	5.0 ± 1.1	4.2 ± 0.56	0.317
Cardiac index (L/min/m^2^)	4.7 ± 1.4	4.6 ± 1.0	4.1 ± 0.6	0.402
RV EDVI (mL/m^2^)	89.0 ± 11.4	84.9 ± 13.7	83.2 ± 6.6	0.609
RV ESVI (mL/m^2^)	31.4 ± 7.1	27.6 ± 5.3	32.8 ± 6.7	0.168
RV mass I (g/m^2^)	19.5 ± 7.7	18.8 ± 3.1	20.3 ± 3.9	0.774
RV EF (%)	64.6 ± 5.6	67.0 ± 4.8	60.2 ± 7.1	0.122

SCD = sickle cell disease, TM = thalassemia major, *N* = number, BMI = body mass index, BSA = body surface area, LV = left ventricular, EDVI = end-diastolic volume index, ESVI = end-systolic volume index, SVI = stroke volume index, EF = ejection fraction, RV = right ventricular. Statistical methods: Kruskal–Wallis test.

## Data Availability

The original contributions presented in this study are included in the article. Further inquiries can be directed to the corresponding author.
